# Supplementary material for: S-Trimer, a COVID-19 subunit vaccine candidate, induces protective immunity in nonhuman primates
Source: Nat Commun. 2021 Mar 1;12:1346. doi: 10.1038/s41467-021-21634-1 (PMC7921634; doi:10.1038/s41467-021-21634-1)
Supplement: Supplementary file 1 — Supplementary Information [file 41467_2021_21634_MOESM1_ESM.pdf]

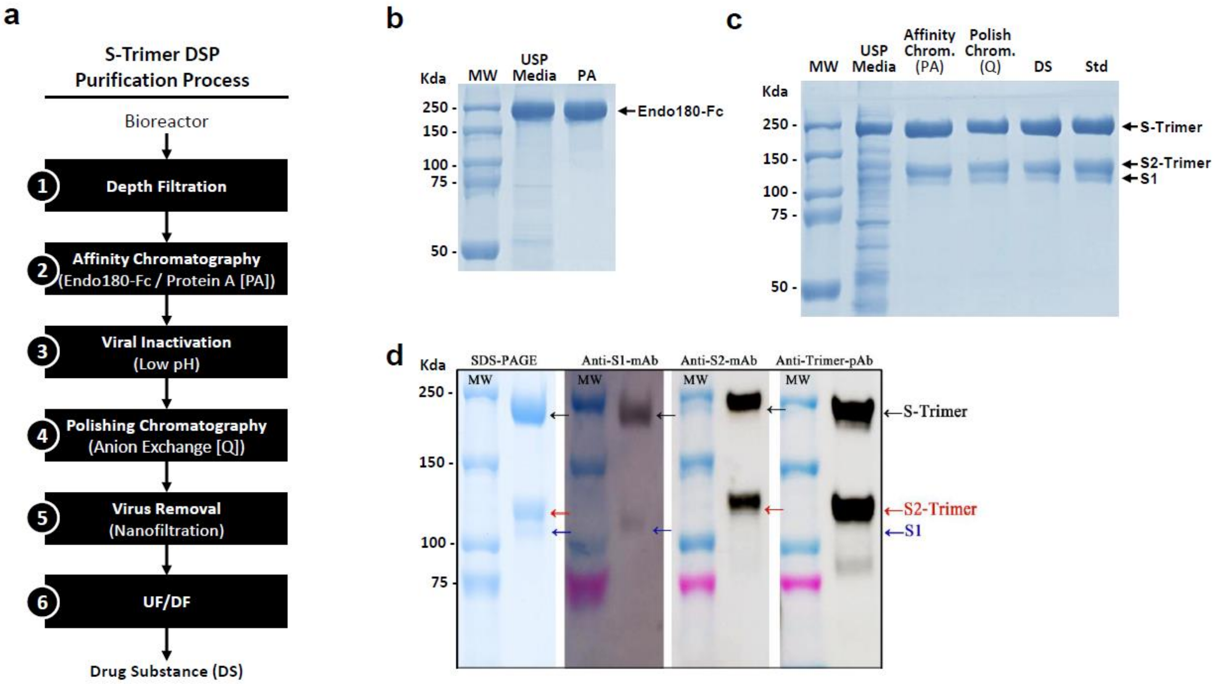

Fig. S1. Purification process of S-Trimer. (a) Downstream purification (DSP) process flow diagram for S-Trimer. **(b)** Non-reducing SDS-PAGE analysis of Endo180-Fc before and after purification from upstream process development (USP) serum-free media using a Protein A chromatography. **(c)** Reducing SDS-PAGE analysis of S-Trimer before and after purification from serum-free media via Endo-180-Fc/ Protein A chromatography (PA), anion-exchange chromatography (Q) and final UF/DF to obtain vaccine drug substance (DS). Reference standard was loaded as a control. **(d)** 0.2 µg of purified protein was analyzed by Western blot under reducing condition using monoclonal antibody against S1 and S2 domain, respectively and a polyclonal antibody against Trimer, uncropped blots in Source Data. The data shown are representative of at least two independent experiments.

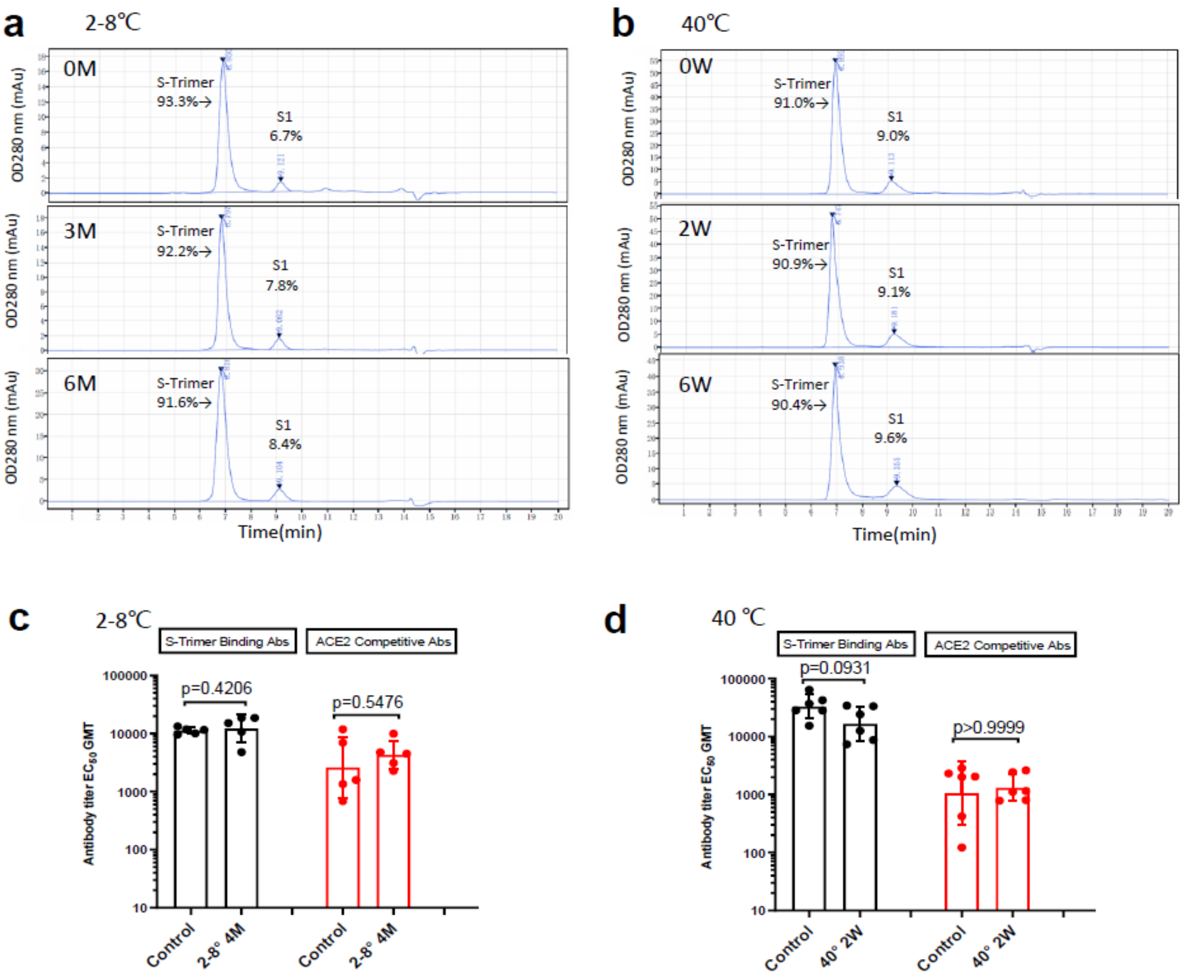

Fig. S2. Stability of S-Trimer antigen in formulation buffer. SEC-HPLC analysis of the purity and integrity of representative lots of S-Trimer after storing for extended time as indicated at 2-8℃ **(a)** and 40℃ **(b)**, respectively. M represents month, and W represents week. Immunogenicity analysis (S-Trimer binding antibody ELISA and ACE2-competitive ELISA) of the stability of S-Trimer after storing at 2-8℃ for 4 month **(c)** and at 40℃ for 2 weeks **(d)**. BALB/c mice (n=5-6/group) were immunized twice, 3 weeks apart intramuscularly (IM) with 3μg of S-Trimer plus AS03 adjuvant. Anti sera where analyzed 2 weeks after 2nd immunization. Data were presented as geometric mean titers (GMT)± SD of EC_{50} for each group and the points represent individual animals. Two-tailed Mann-Whitney tests were used to compare the two experiment groups. P values < 0.05 were considered significant.

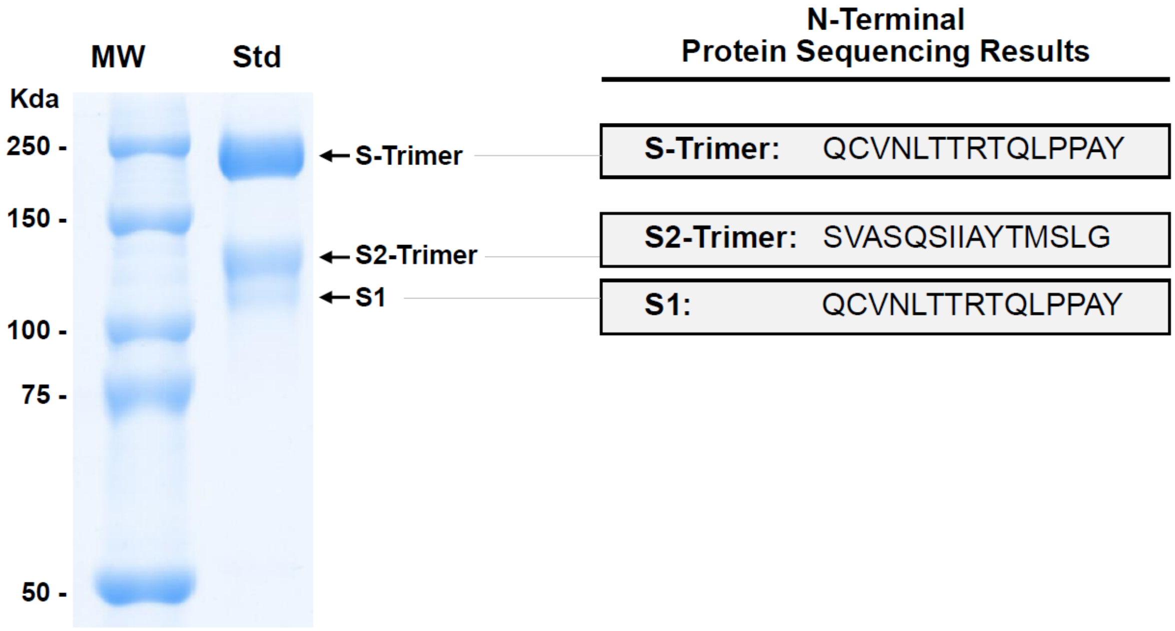

Fig. S3. N-terminal peptide sequencing of partially-cleaved S-Trimer. Initial results showed the full-length S-Trimer could not be sequenced directly by Edman degradation method, while S2-Trimer fragment yield peptide sequence shown with predicted furin protease cleavage at S1/S2 boundary. Following the removal of 14Q by pyroglutamate aminopeptidase, the N-terminal sequence for the full-length S-Trimer was determined by subsequent Edman degradation as indicated. Since the S2-Trimer had predicted N-terminus after furin cleavage, the N-terminus of S1 was predicted to be the same as that of full-length S-Trimer. The data shown are representative of two independent experiments.

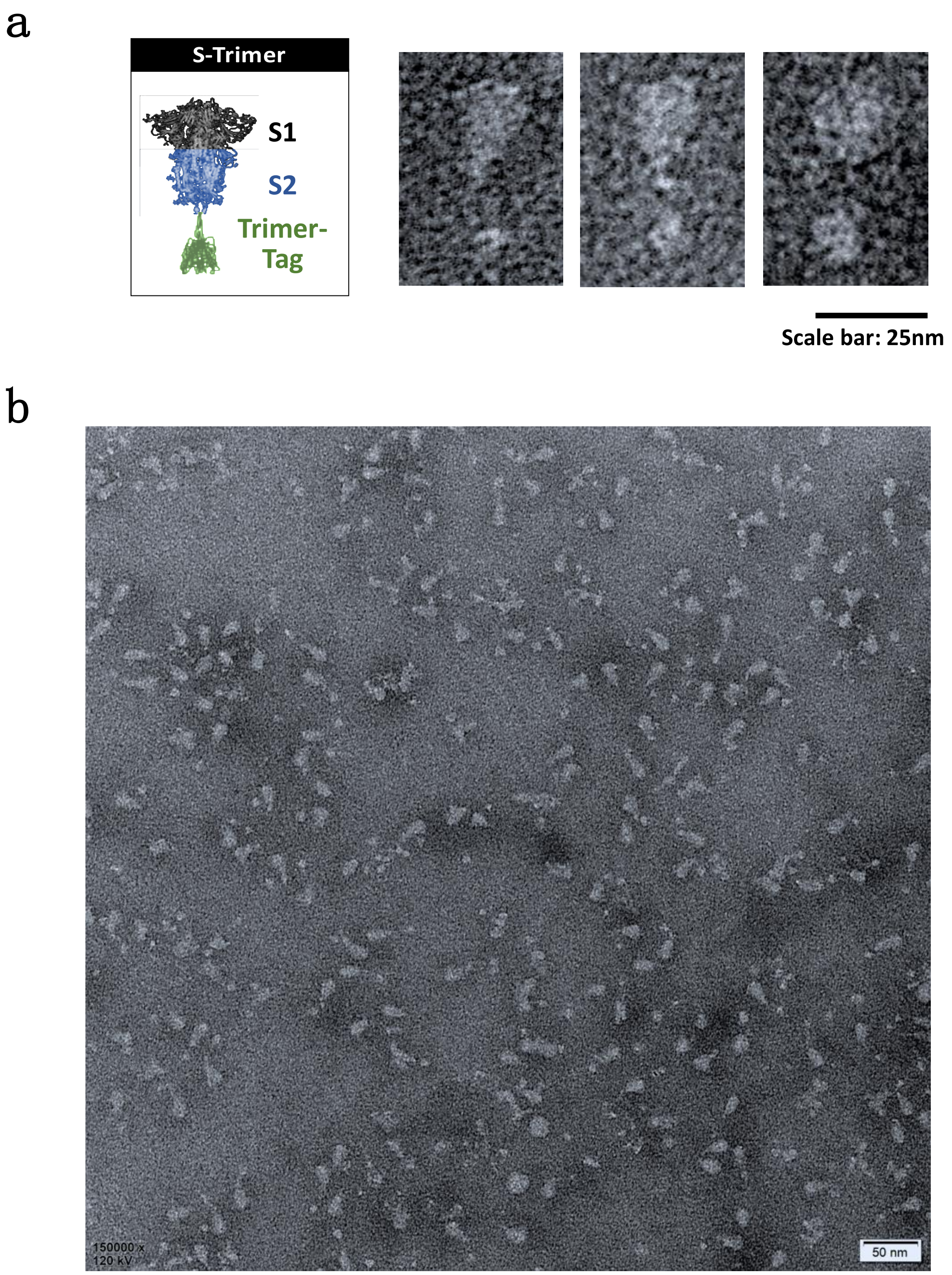

Fig. S4. Negative-stain electron microscopy image of purified S-Trimer. (a) Illustrative structure and representative negative-stain EM images S-Trimer with homotrimeric Spike protein in the prefusion conformation. **(b)** Raw negative-stain EM image and visualization of purified S-Trimer antigens predominantly in the prefusion conformation. Data shown are representative of two independent experiments.

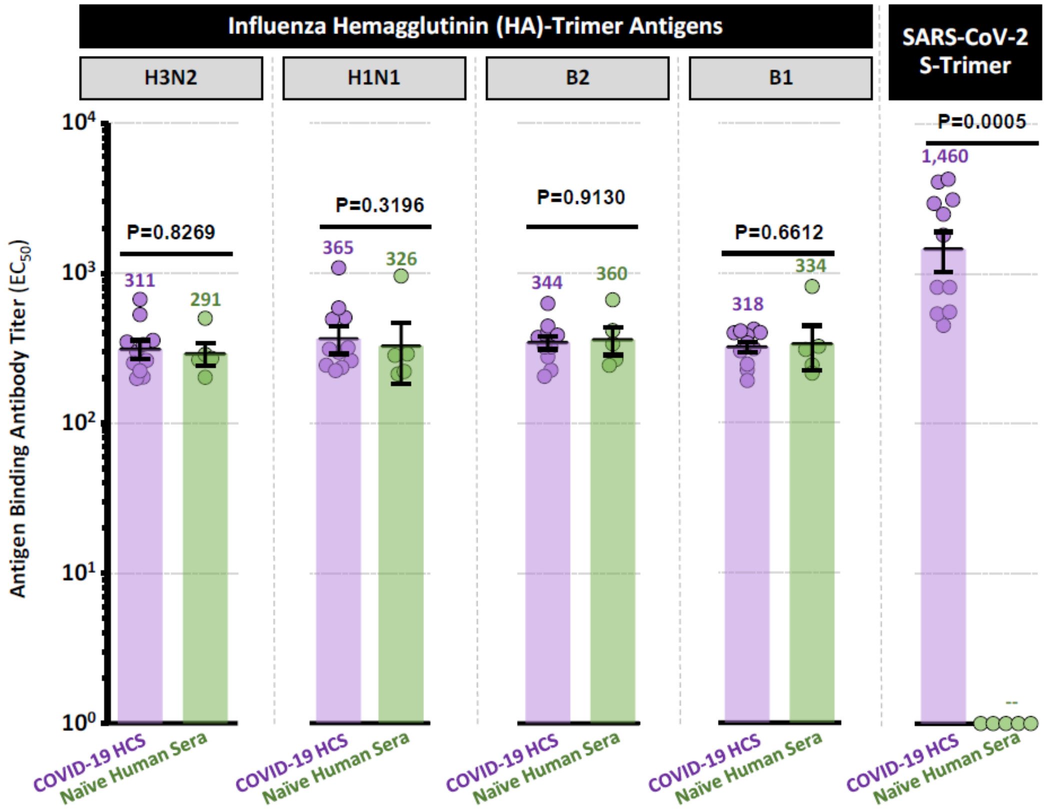

Fig. S5. Specificity of S-Trimer binding antibody ELISA assay. Human convalescent sera collected from recovered COVID-19 subjects (n=11) and naïve human volunteer sera (n=5) and were analyzed for antibodies binding to influenza hemagglutinin (HA)-Trimer antigens (H3N2, H1N1, B2 and B1 strains) or SARS-CoV-2 S-Trimer antigen. Points represent individual humans; horizontal lines indicate geometric mean titers (GMT) ± SEM of EC_{50} for each group. Statistical significance was calculated with a two-tailed Mann-Whitney test. P values < 0.05 were considered significant.

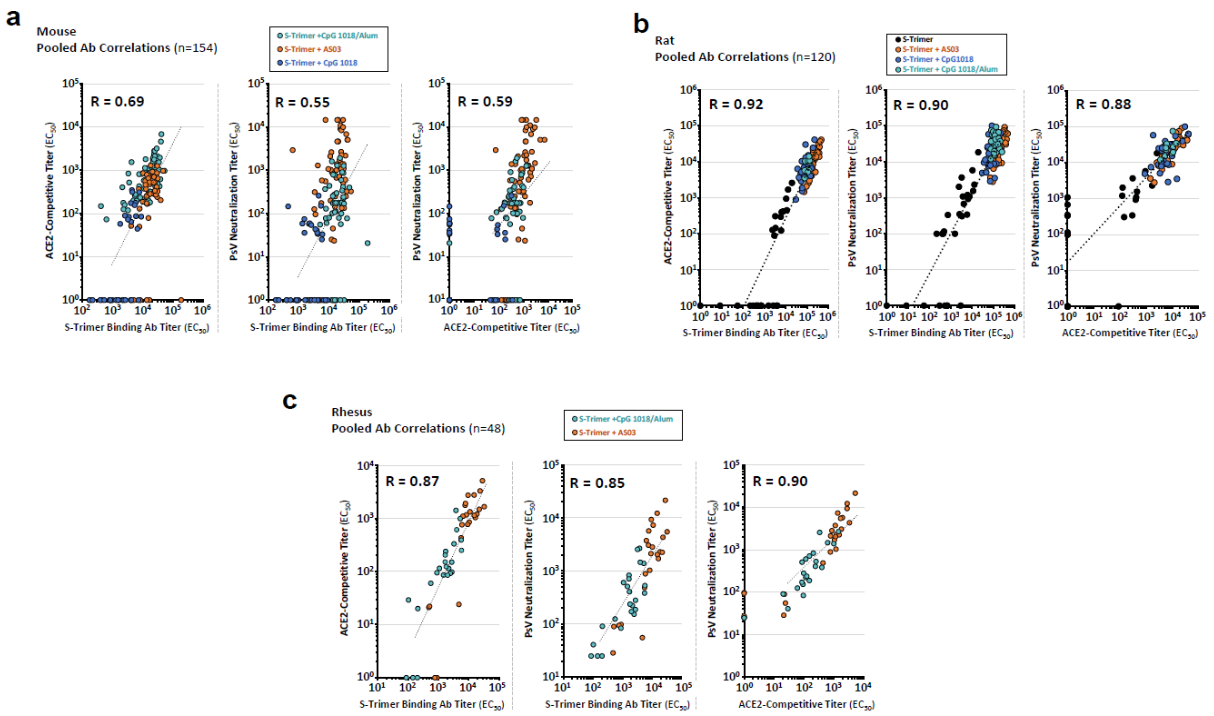

Fig. S6. Correlation of antibody titers in immunized mice, rats and Rhesus Macaques. Antibody titers in **(a)** immunized mice, **(b)** immunized rats, and **(c)** immunized rhesus macaques based on three assays (S-Trimer binding antibodies, ACE2-competitive, and pseudovirus neutralization) were analyzed for correlation based on two-tailed Pearson's R analysis. Data presented are from post-immunization samples at multiple timepoints. Points represent individual animal samples.

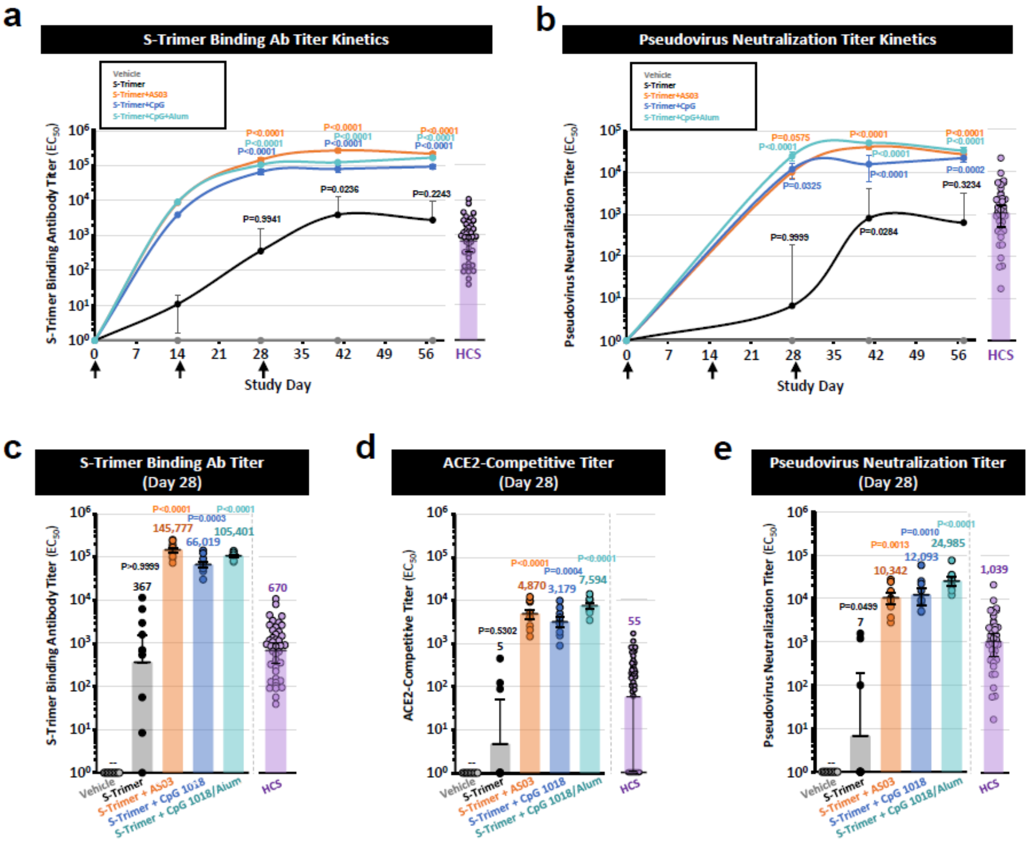

Fig. S7. **Immunogenicity of S-Trimer in SD rats.** SD Rats (n=10/group) were immunized with 30 µg of S-Trimer adjuvanted with PBS vehicle control, 30 µg S-Trimer (non-adjuvanted), 30 µg S-Trimer adjuvanted with 0.25 mL of AS03, 30 µg S-Trimer adjuvanted with 1.5 mg CpG 1018, or 30 µg S-Trimer adjuvanted with 0.75 mg CpG 1018 plus 0.375 mg alum three times on Day 0, Day 14 and Day 28. The humoral immune responses and kinetics were analyzed and compared with a human convalescent sera (HCS) panel, based on **(a)** S-Trimer binding antibody ELISA titers and **(b)** SARS-CoV-2 pseudovirus neutralization titers. Data are presented as geometric mean titers (GMT) ± SEM and the comparisons were made against the vehicle control group with Two-way ANOVA multiple comparisons test. Antibody titers two weeks after the second immunization on Day 28 (prior to the third immunization) are shown for **(c)** S-Trimer binding antibody ELISA titers vs. HCS, **(d)** ACE2-competitive ELISA titers vs. HCS, and **(e)** SARS-CoV-2 pseudovirus neutralization titers vs. HCS. Points represent individual animals and humans; horizontal lines indicate GMT of EC_{50} for each group and Kruskal-Wallis ANOVA with Dunn's multiple comparisons test was applied. P values < 0.05 were considered significant.

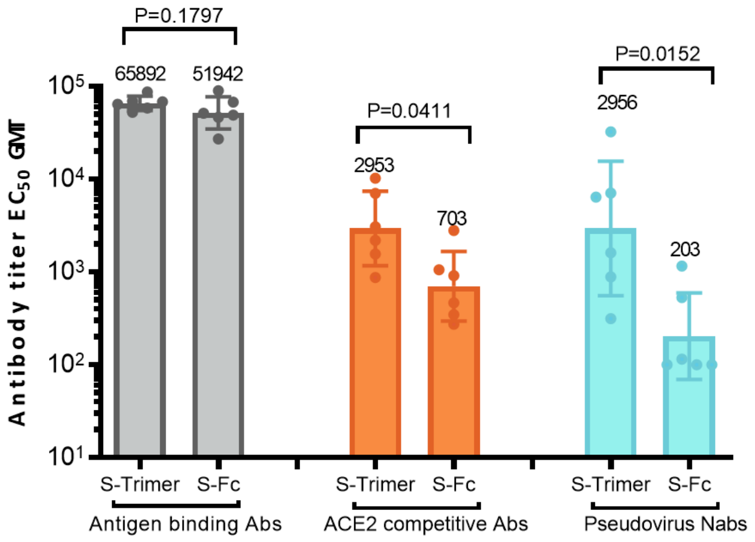

Fig. S8. Side-by-side comparison of immunogenicity of S-Trimer and S-Fc in Mice. BALB/c mice (n=6/group) were immunized intramuscularly (IM) with either 3µg of S-Trimer or S-Fc adjuvanted with 25 µL of AS03 (GSK). The total injection volume of the mixed vaccines (antigen + adjuvant) was 50 µL per IM dose. Two IM doses were administered (at Day 0 and Day 21). Animals were bled from the tail veins for humoral immune response analysis, including Antigen binding antibody ELISA titers (left), ACE2-competitive ELISA titers (middle), and SARS-CoV-2 pseudovirus neutralization titers (right) on day 35. Data were presented as geometric mean titers (GMT) ± SD, and the points represent individual animals. Statistical significance was calculated with a two-tailed Mann-Whitney test. P values < 0.05 were considered significant.

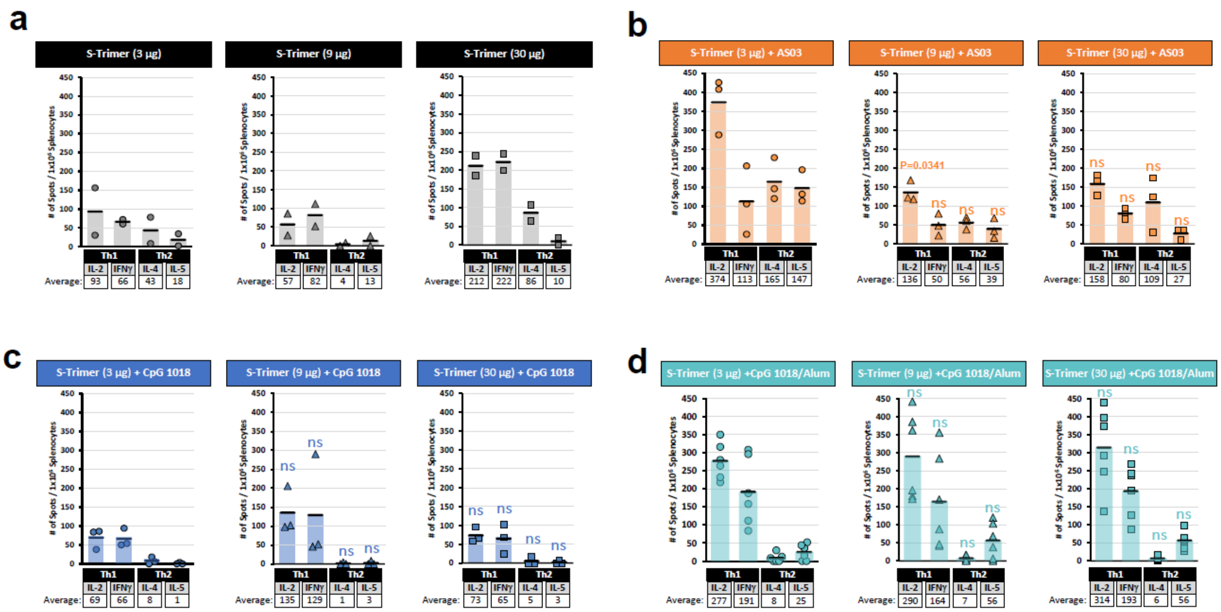

Fig. S9. Th1 and Th2 cell-mediated immune response of S-Trimer in mice by ELISpot. BALB/c mice (n=7-8/group) were immunized with various doses of S-Trimer that was non-adjuvanted or adjuvanted with AS03, CpG 1018-alone, or CpG 1018 plus alum twice on Day 0 and Day 21. After necropsy, splenocytes were harvested from mice and stimulated with S-Trimer antigen, followed by detection of Th1 (IL-2, IFNγ) and Th2 (IL-4, IL-5) cytokines by ELISpot for the **(a)** non-adjuvanted S-Trimer groups(n=2), **(b)** AS03-adjuvanted S-Trimer groups(n=3), **(c)** CpG 1018-adjuvanted S-Trimer groups(n=3), and **(d)** CpG 1018 plus alum adjuvanted S-Trimer groups(n=6). Points represent individual animals; horizontal lines indicate mean values for each group. For each cytokine, readouts from all dosages were compared to that of 3µg with Kruskal-Wallis ANOVA with Dunn's multiple comparisons test. P values < 0.05 were considered significant. ns, no significance.

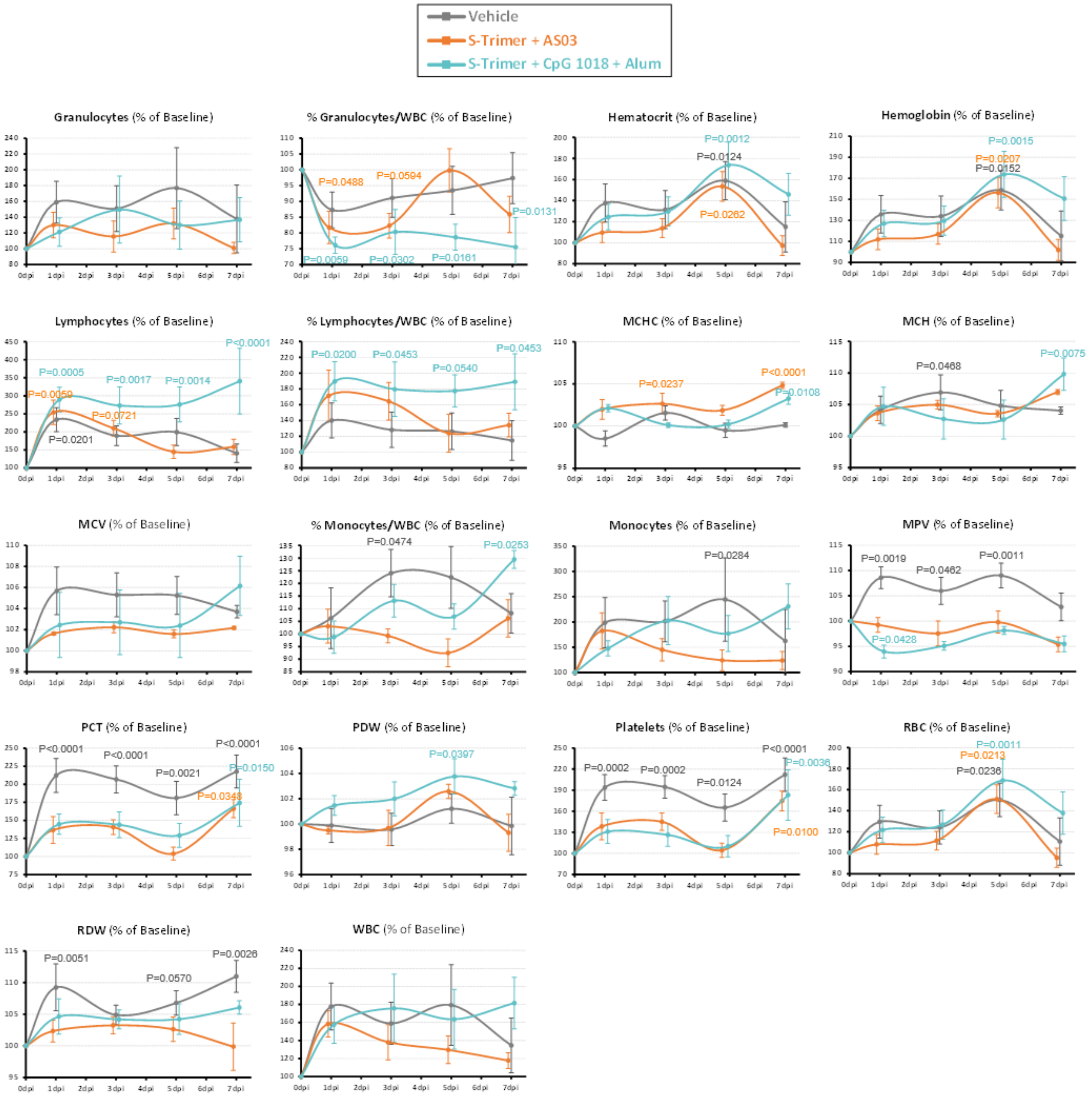

Fig. S10. Change in hematology parameters in Rhesus Macaques after SARS-CoV-2 challenge. Rhesus macaques (n=6/group) were immunized with 30 µg S-Trimer adjuvanted with 0.25 mL AS03, 30 µg S-Trimer adjuvanted with 1.5 mg CpG 1018 plus 0.75 mg alum, or PBS vehicle control twice on Day 0 and Day 21 and were challenged on Day 35 with 2.6 x 10^6 TCID50 (60% intratracheal and 40% intranasal) live SARS-CoV-2 virus. Following SARS-CoV-2 challenge, various blood chemistry parameters were analyzed at 0, 1, 3, 5 and 7 dpi. Data are presented as mean ± SEM. All comparisons were made against the corresponding values on 0 dpi with Two-way ANOVA multiple comparisons test. P values < 0.05 were considered significant and shown in the figure.

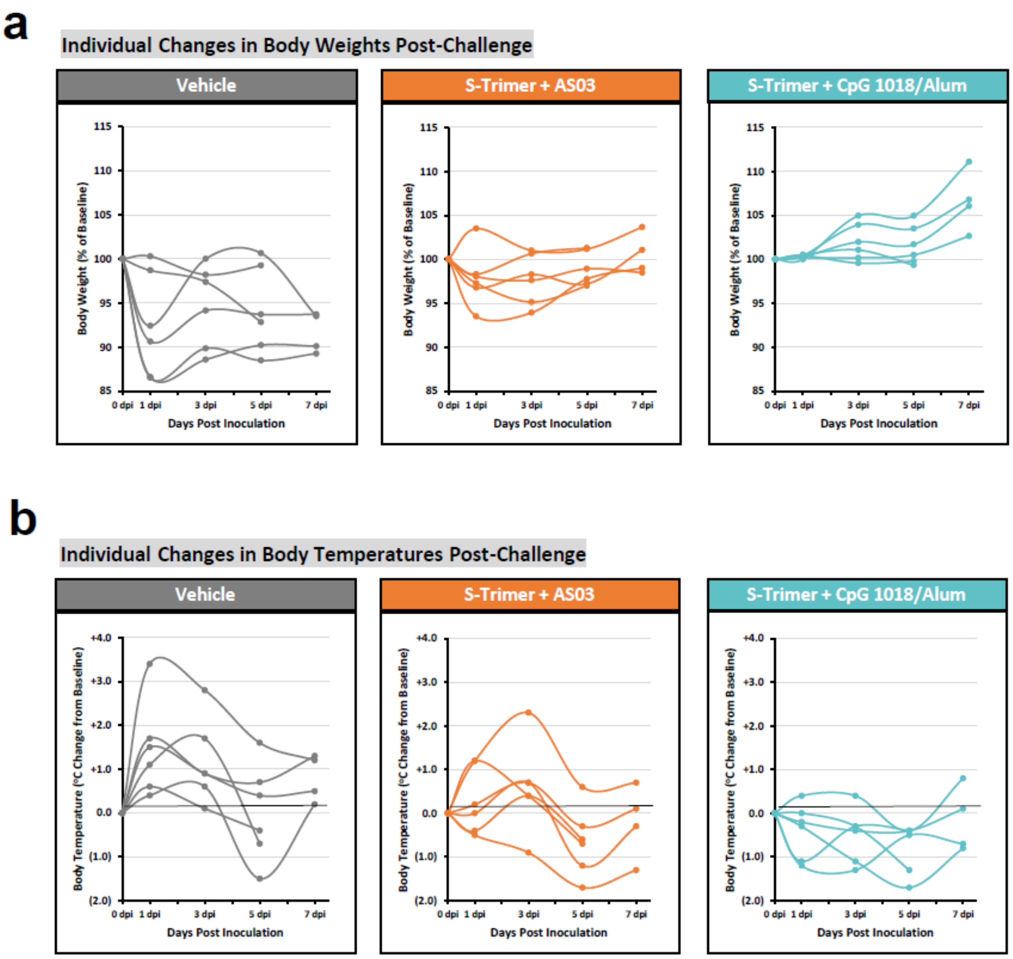

Fig. S11. Change in individual body weights and body temperatures in Rhesus Macaques after SARS-CoV-2 challenge. Rhesus macaques (n=6/group) were immunized with 30 µg S-Trimer adjuvanted with 0.25 mL AS03, 30 µg S-Trimer adjuvanted with 1.5 mg CpG 1018 plus 0.75 mg alum, or PBS vehicle control twice on Day 0 and Day 21 and were challenged on Day 35 with 2.6 x 10^6 TCID50 (60% intratracheal and 40% intranasal) live SARS-CoV-2 virus. Following SARS-CoV-2 challenge, clinical observation data were collected based on **(a)** changes in body weight and **(b)** changes in body temperature at 0, 1, 3, 5 and 7 dpi. Individual animal data are presented.

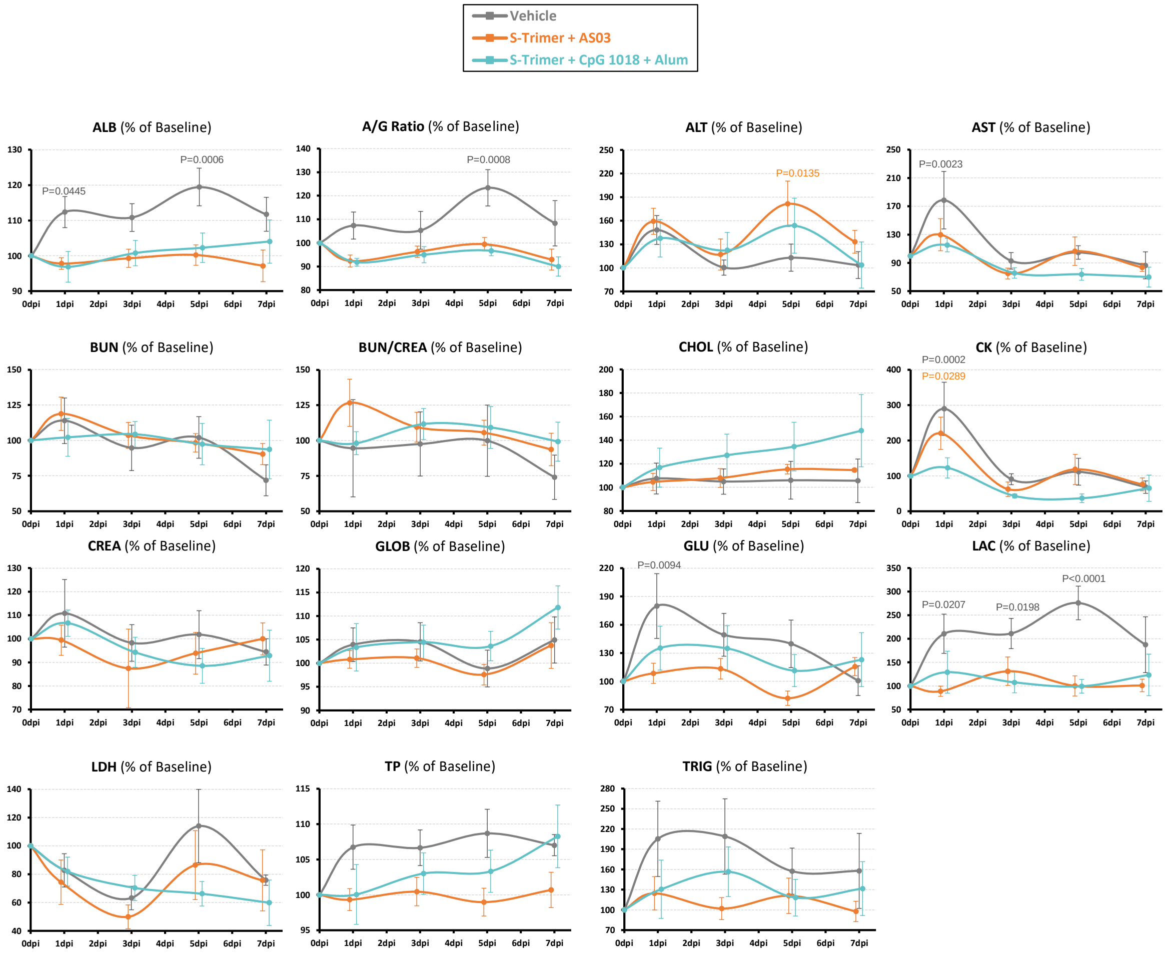

Fig. S12. Change in blood chemistry parameters in Rhesus Macaques after SARS-CoV-2 challenge. Rhesus macaques (n=6/group) were immunized with 30 µg S-Trimer adjuvanted with 0.25 mL AS03, 30 µg S-Trimer adjuvanted with 1.5 mg CpG 1018 plus 0.75 mg alum, or PBS vehicle control twice on Day 0 and Day 21 and were challenged on Day 35 with 2.6 x 10^6 TCID50 (60% intratracheal and 40% intranasal) live SARS-CoV-2 virus. Following SARS-CoV-2 challenge, various hematology parameters were analyzed at 0, 1, 3, 5 and 7 dpi. Data were presented as mean ± SEM. All comparisons were made against the corresponding values on 0 dpi with Two-way ANOVA multiple comparisons test. P values < 0.05 were considered significant and shown in the figure.

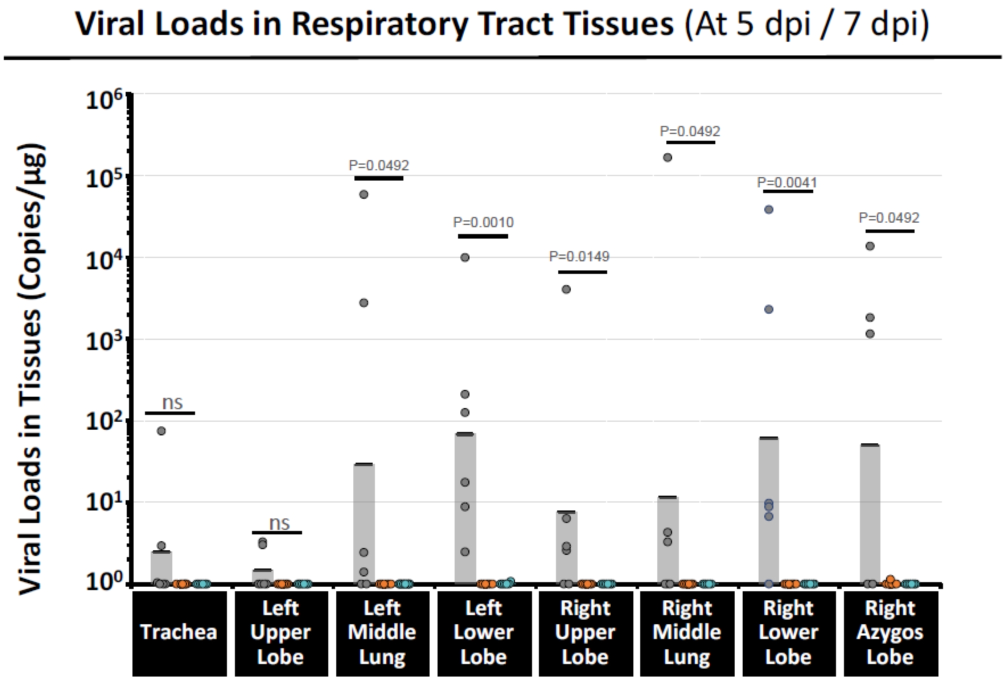

% of Animals With Detectable Viral Loads in:	≥ 3 Tissues	≥ 6 Tissues
Vehicle	*83%* (5/6)	*50%* (3/6)
S-Trimer + AS03	--	--
S-Trimer + CpG 1018/Alum	--	--

Fig. S13. Viral loads in lung tissues in Rhesus Macaques at necropsy following SARS-CoV-2 challenge. Rhesus macaques (n=6/group) were immunized with 30 µg S-Trimer adjuvanted with 0.25 mL AS03, 30 µg S-Trimer adjuvanted with 1.5 mg CpG 1018 plus 0.75 mg alum, or PBS vehicle control twice on Day 0 and Day 21 and were challenged on Day 35 with 2.6 x 10^6 TCID50 (60% intratracheal and 40% intranasal) live SARS-CoV-2 virus. Following SARS-CoV-2 challenge and after necropsy at 5 dpi (n=2/group) and 7 dpi (n=4/group), various lung and trachea tissues were collected for measurement of viral loads based on genomic RNA (gRNA). Points represent individual animals; horizontal lines indicate geometric mean values for each group. All comparisons were made against the vehicle control group with Kruskal-Wallis ANOVA with Dunn’s multiple comparisons test. P values < 0.05 were considered significant. ns, no significance.

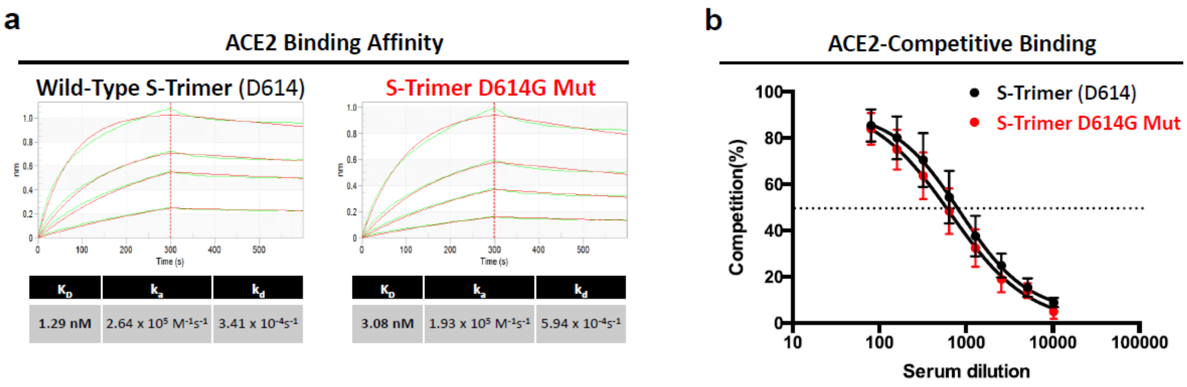

Fig. S14. Comparison of ACE2-binding for S-Trimer (D614) and S-Trimer D614G Mut. (a) Determination of the binding affinity between wild-type S-Trimer (D614) or S-Trimer D614G mutant and human ACE2-Fc by Fortebio biolayer interferometry. **(b)** Determination of the ACE2-competitive binding by ELISA of wild-type S-Trimer (D614) or S-Timer D614G mutant with sera collected from mice immunized with two IM doses of wild-type S-Trimer adjuvanted with AS03 (n=6/experiment). Data were presented as mean ± SEM.

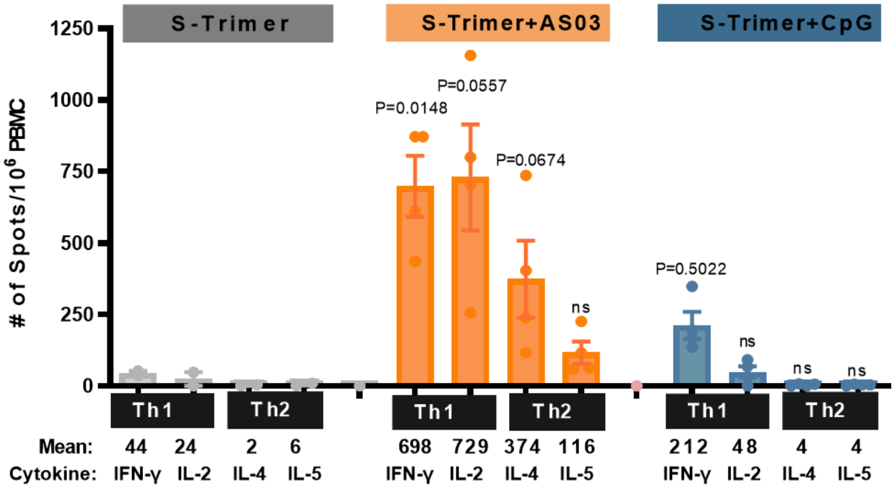

Fig. S15. Th1 and Th2 cell-mediated immune response of S-Trimer in Nonhuman Primates by ELISpot. Nonhuman Primates (n=2-4/group) were immunized with 25μg S-Trimer that was non-adjuvanted or adjuvanted with either AS03 or CpG 1018-alone on Day 0 and Day 21. PBMC were isolated from NHP on day 35, and stimulated with S-Trimer antigen, followed by detection of Th1 (IL-2, IFNγ) and Th2 (IL-4, IL-5) cytokines by ELISpot for the non-adjuvanted S-Trimer groups (n=2), AS03-adjuvanted S-Trimer groups (n=4) and CpG 1018-adjuvanted S-Trimer groups (n=4). Points represented individual animals. Data were mean ± SEM. For all cytokines, the comparisons were compared to groups vaccinated without adjuvant. Kruskal-Wallis ANOVA with Dunn's multiple comparisons test was applied. P values < 0.05 were considered significant. ns, no significance.

	Age (years)	Gender (Male/Female)		S-Trimer Binding Ab (EC50)	ACE2-Competitive (EC50)	PsV NAb (EC50)
MILD COVID-19 Disease (n=10)	65	Male		4,409	--	2,133
	63	Female		1,326	494	2,291
	67	Female		851	99	1,448
	41	Male		320	74	893
	62	Female		222	n.d.	58
	66	Male		151	n.d.	341
	25	Male		122	n.d.	280
	27	Female		93	n.d.	90
	34	Male		91	n.d.	917
	33	Female		39	n.d.	56
**Avg:**	**48.3**	**50% M / 50% F**	**GMT:**	**271**	**5**	**418**
MODERATE COVID-19 Disease (n=17)	54	Female		4,173	197	3,018
	59	Female		3,313	173	2,800
	55	Male		2,615	637	2,197
	41	Male		2,586	188	1,117
	62	Female		1,605	781	6,027
	74	Female		1,463	121	642
	57	Female		1,202	163	455
	59	Male		937	208	970
	49	Female		901	90	5,413
	47	Male		830	782	1,760
	47	Female		628	152	670
	50	Male		409	101	182
	23	Male		271	n.d.	1,136
	55	Female		135	82	1,953
	35	Male		127	n.d.	1,242
	36	Female		102	n.d.	185
	30	Female		56	n.d.	519
**Avg:**	**49.0**	**41% M / 59% F**	**GMT:**	**675**	**59**	**1,151**
SEVERE COVID-19 Disease (n=7)	47	Male		10,471	1,628	5,504
	60	Male		2,999	383	3,996
	37	Male		2,330	598	2,209
	65	Female		1,980	1,083	9,163
	74	Female		1,166	287	4,885
	37	Male		641	235	2,156
	47	Male		352	58	20,804
**Avg:**	**52.4**	**71% M / 29% F**	**GMT:**	**1,682**	**398**	**5,161**
Patient Information Not Available (n=7)	--	--		7,907	340	879
	--	--		1,824	708	5,444
	--	--		1,132	188	391
	--	--		1,066	247	1,536
	--	--		870	336	1,419
	--	--		547	232	399
	--	--		90	n.d.	17

Table S1. Human convalescent sera panel. Characteristics and information of 41 COVID-19 patients, from whom convalescent sera was collected and included for testing in this study. COVID-19 disease severity, patient age and patient gender are included, as well as S-Trimer binding antibody titers (EC_{50}), ACE2-competitive titers (EC_{50}) and SARS-CoV-2 pseudovirus neutralization titers (EC_{50}).

Pearson's R	ACE2-Competitive Titer vs. S-Trimer Binding Ab Titer	PsV Neutralization Titer vs. S-Trimer Binding Ab Titer	PsV Neutralization Titer vs. ACE2-Competitive Titer
**Mice** – BALB/c (n=154)	R = 0.69	R = 0.55	R = 0.59
**Rats** – SD (n=120)	R = 0.92	R = 0.90	R = 0.88
**Monkey** – Rhesus (n=48)	R = 0.87	R = 0.85	R = 0.90
**Human Convalescent Sera** (n=41)	R = 0.86	R = 0.61	R = 0.67

Table S2. Correlation of antibody titers in immunized mice, rats, rhesus and human convalescent sera. Antibody titers in human convalescent sera and in immunized mice, rats, and rhesus macaques based on three assays (S-Trimer binding antibodies, ACE2-competitive, and pseudovirus neutralization) were analyzed for correlation based on two-tailed Pearson's R analysis.

Primer name	Sequence
S-Trimer D614G mutation forward	5’- GCCGTGCTGTATCAGGGCGTGAATTGTACCGAG -3’
S-Trimer D614G mutation reverse	5’- CTCGGTACAATTCACGCCCTGATACAGCACGGC -3’
SARS-COV-2 N forward	5’-GGGGAACTTCTCCTGCTAGAAT-3’
SARS-COV-2 N reverse	5’-CAGACATTTTGCTCTCAAGCTG-3’
SARS-COV-2 N probe	5’-FAM-TTGCTGCTGCTTGACAGATT-TAMRA-3’

Table S3. Primers used for this study.
