# Supplementary material for: S-Trimer, a COVID-19 subunit vaccine candidate, induces protective immunity in nonhuman primates
Source: Nat Commun. 2021 Mar 1;12:1346. doi: 10.1038/s41467-021-21634-1 (PMC7921634; doi:10.1038/s41467-021-21634-1)
Supplement: Supplementary file 3 — Reporting Summary [file 41467_2021_21634_MOESM3_ESM.pdf]

nature research

Corresponding author(s): Peng Liang

Last updated by author(s): Jan 27, 2021

Reporting Summary

Nature Research wishes to improve the reproducibility of the work that we publish. This form provides structure for consistency and transparency in reporting. For further information on Nature Research policies, see our Editorial Policies and the Editorial Policy Checklist.

Statistics

For all statistical analyses, confirm that the following items are present in the figure legend, table legend, main text, or Methods section.

n/a	Confirmed	
☐	☒	The exact sample size (*n*) for each experimental group/condition, given as a discrete number and unit of measurement
☐	☒	A statement on whether measurements were taken from distinct samples or whether the same sample was measured repeatedly
☐	☒	The statistical test(s) used AND whether they are one- or two-sided<br>*Only common tests should be described solely by name; describe more complex techniques in the Methods section.*
☒	☐	A description of all covariates tested
☐	☒	A description of any assumptions or corrections, such as tests of normality and adjustment for multiple comparisons
☐	☒	A full description of the statistical parameters including central tendency (e.g. means) or other basic estimates (e.g. regression coefficient) AND variation (e.g. standard deviation) or associated estimates of uncertainty (e.g. confidence intervals)
☐	☒	For null hypothesis testing, the test statistic (e.g. *F*, *t*, *r*) with confidence intervals, effect sizes, degrees of freedom and *P* value noted<br>*Give P values as exact values whenever suitable.*
☒	☐	For Bayesian analysis, information on the choice of priors and Markov chain Monte Carlo settings
☒	☐	For hierarchical and complex designs, identification of the appropriate level for tests and full reporting of outcomes
☐	☒	Estimates of effect sizes (e.g. Cohen's *d*, Pearson's *r*), indicating how they were calculated

Our web collection on statistics for biologists contains articles on many of the points above.

Software and code

Policy information about availability of computer code

Data collection	1.The binding affinity data was collected by Bio-Layer Interferometry measurements on ForteBio Octet QKe (Pall), OCTET data acquisition 12.0<br>2.ELISA data were collected using Multiskan Go microplate reader （Thermo Fisher）<br>3.pseudovirus neutralization assay data were collected using fluorescence microplate （TECAN, Spark）<br>4.Pathologic slides were digitized by upright microscope (BX53, Olympus)<br>5.The purity of S-Trimer was analyzed by Size-Exclusion Chromatography (SEC-HPLC) using Agilent 1260 Infinity HPLC with an analytic TSK gel G3000 SWxL column (Tosoh)
Data analysis	Prism 8.0 (GraphPad Software)<br>origin 9<br>Excel O365<br>OCTET data analysis HT12.0

For manuscripts utilizing custom algorithms or software that are central to the research but not yet described in published literature, software must be made available to editors and reviewers. We strongly encourage code deposition in a community repository (e.g. GitHub). See the Nature Research guidelines for submitting code & software for further information.

Data

Policy information about availability of data

All manuscripts must include a data availability statement. This statement should provide the following information, where applicable:

- Accession codes, unique identifiers, or web links for publicly available datasets
- A list of figures that have associated raw data
- A description of any restrictions on data availability

No genomic or microarray data sets were generated in this study. Reference SARS-CoV-2 Spike protein and ACE2 sequences were downloaded from the NCBI database (GenBank: MN908947.3 and NM_001371415.1), HA protein sequences were downloaded from EpiFluDatabase (Accession Nos: EPI516535, EPI614444, EPI540675, EPI498048). Source data are provided with this paper.

Field-specific reporting

Please select the one below that is the best fit for your research. If you are not sure, read the appropriate sections before making your selection.

[x] Life sciences [ ] Behavioural & social sciences [ ] Ecological, evolutionary & environmental sciences

For a reference copy of the document with all sections, see nature.com/documents/nr-reporting-summary-flat.pdf

Life sciences study design

All studies must disclose on these points even when the disclosure is negative.

Sample size	Sample sizes were determined based on our and other investigators experience. No statistical methods were used to predetermine sample size. Balb/c mouse immunogenicity study(n=6-8 for each group, Dai et al, Cell 2020); SD rat(n=10, half male and female, Wang et al, Cell 2020); Rhesus(n=6 for each group, Wang et al, Cell 2020; Gao et al, Science 2020)
Data exclusions	No data were excluded from the analysis
Replication	Replicates were used in the experiments as noted in the figure legends.
Randomization	Female Mice of 6- to 8-week old were randomly assigned to different treatment groups, SD rats (6-9 weeks old) were balanced for age and gender and otherwise randomly allocated to groups. male rhesus 3-6 years of age were randomly assigned to different treatment groups.
Blinding	Researchers were not blinded in the process of experiments or data analysis since all of the findings are supported by quantitative measurement.

Reporting for specific materials, systems and methods

We require information from authors about some types of materials, experimental systems and methods used in many studies. Here, indicate whether each material, system or method listed is relevant to your study. If you are not sure if a list item applies to your research, read the appropriate section before selecting a response.

Materials & experimental systems

n/a	Involved in the study	
[ ]	[x]	Antibodies
[ ]	[x]	Eukaryotic cell lines
[x]	[ ]	Palaeontology and archaeology
[ ]	[x]	Animals and other organisms
[ ]	[x]	Human research participants
[x]	[ ]	Clinical data
[x]	[ ]	Dual use research of concern

Methods

n/a	Involved in the study	
[x]	[ ]	ChIP-seq
[x]	[ ]	Flow cytometry
[x]	[ ]	MRI-based neuroimaging

Antibodies

Antibodies used	ELISA:Goat anti-mouse IgG ads HRP(Southern-biotech, 1031-05); Goat anti-HUMAN IgG ads HRP(Southern-biotech, 2081-05); Goat anti-rabbit IgG ads HRP (Southern-biotech CAT:4010-05);Rabbit anti monkey-IgG-HRP (solarbio SE241); goat anti-Rat IgG-HRP ((Southern-biotech, 3051-05)<br>Elispot: Mouse IL-2 ELISpotPLUS kit Mabtech 3441-4APW-2; Mouse IL-4 ELISpotPLUS Mabtech 3311-4APW-2; Mouse IL-5 ELISpotPLUS kit Mabtech 3391-4APW-2; Mouse IFN-γ ELISpotPLUS Mabtech 3321-4AST-2<br>IHC: mouse monoclonal antibody specific to S1 of SARS-CoV-2 (Clover Biopharma) ;HRP-conjugated secondary antibody (ZSGB Bio PV-6002)

	WB:mouse monoclonal antibody specific to S1 and S2 of SARS-CoV-2 (Clover Biopharma) ; rabbit antibody specific to trimer (Clover Biopharma)
Validation	The primary antibodies used in this study were S1 and S2 of SARS-CoV-2 mouse mAb and rabbit anti-Trimer pAbs, the 3 antibodies were produced in Clover Biopharma and validated.<br>S1 mAb was validated by Elisa on S1 fusion protein.<br>S2 mAb was validated by Elisa SARS-CoV-2 on S and S1 fusion protein.<br>Trimer-Tag pAb was validated by Elisa and WB on Trimer-Tag fusion protein.

Eukaryotic cell lines

Policy information about cell lines

Cell line source(s)	GH-CHO(DHFR-/-) was obtained from GenHunter Corporation; Vero E6 was obtained from KangHua Biological Product CO.,LTD; HEK-293F was obtained from Clover Biopharma.
Authentication	Cell lines were authenticated by morphology
Mycoplasma contamination	Neither of the cell lines used in this study tested positive for Mycoplasma
Commonly misidentified lines<br>(See ICLAC register)	no commonly misidentified cell lines were used in the study.

Animals and other organisms

Policy information about studies involving animals; ARRIVE guidelines recommended for reporting animal research

Laboratory animals	Specific pathogen-free (SPF) BALB/c female mice (6-8 weeks old) for immunogenicity studies were purchased from Chengdu Dossy Experimental Animals Co., LTD ;SD rats (6-9 weeks old, half male and female) were purchased from Zhejiang Vital River Laboratory Animal Technology Co., Ltd. The Rhesus Macaque(3-6 years old, male)SARS-CoV-2 challenge study was performed at Kunming Institute of Zoology.
Wild animals	no wild animals were used in the study.
Field-collected samples	no field collected samples were used in the study.
Ethics oversight	All mouse experiments were approved by the institutional animal care and use committee (IACUC) in Clover Biopharmaceutical and were conducted according to international guidelines for animal studies.Studies with SD rats were compliant with the policies of JOINN Laboratories Inc., the Guide for the Care and Use of Laboratory Animals (8th Edition, Institute of Laboratory Animal Resources, Commission on Life Sciences, National Research Council; National Academy Press; Washington, D.C., 2010), and the U.S. Department of Agriculture through the Animal Welfare Act (Public Law 99-198). The Rhesus Macaque SARS-CoV-2 challenge study was performed at Kunming Institute of Zoology, Chinese Academy of Sciences (CAS),The biosafety level 3 (BSL3) lab at CAS followed the international guidelines for the animal experiment and approved by the institutional animal ethics committee of KIZ, CAS (No.: IACUC20005) prior to the studies.

Note that full information on the approval of the study protocol must also be provided in the manuscript.

Human research participants

Policy information about studies involving human research participants

Population characteristics	41 human convalescent sera samples from recovered COVID-19 patients were obtained from Public Health Clinical Center of Chengdu in Chengdu, China. The age and gender information were supplied in Table S1.
Recruitment	This study is not a part of clinical trial. The serum samples from 41 patients that were infected by the virus during the early 2020 outbreak in china.
Ethics oversight	41 human convalescent sera samples from recovered COVID-19 patients were obtained from Public Health Clinical Center of Chengdu in Chengdu, China, under approved guidelines by the Institutional Review Board (IRB), and all patients had provided written informed consent before sera sample were collected.

Note that full information on the approval of the study protocol must also be provided in the manuscript.
